# Identification of Immune-Related Therapeutically Relevant Biomarkers in Breast Cancer and Breast Cancer Stem Cells by Transcriptome-Wide Analysis: A Clinical Prospective Study

**DOI:** 10.3389/fonc.2020.554138

**Published:** 2021-02-24

**Authors:** Linbang Wang, Wei Liu, Jingkun Liu, Yuanyuan Wang, Jiaojiao Tai, Xuedong Yin, Jinxiang Tan

**Affiliations:** ^1^ Department of Orthopedic Surgery, The First Affiliated Hospital of Chongqing Medical University, Chongqing, China; ^2^ Department of Orthopedics, Honghui Hospital, Xi’an Jiaotong University, Xi’an, China; ^3^ Department of Endocrine and Breast Surgery, The First Affiliated Hospital of Chongqing Medical University, Chongqing, China

**Keywords:** breast cancer, cancer stem cell, tumor immune infiltration, *PIMREG*, *MTFR2*

## Abstract

Cancer stem cells (CSCs) represent a subset of tumor cells that are responsible for recurrence and metastasis of tumors. These cells are resistant to radiotherapy and chemotherapy. Immunotherapeutic strategies that target CSCs specifically have provided initial results; however, the mechanism of action of these strategies is unclear. The data were requested from The Cancer Genome Atlas and Genotype-Tissue Expression, followed with the survival analysis and weighted gene co-expression network analysis to detect survival and stemness related genes. Patients were divided into three groups based on their immune status by applying single sample GSEA (ssGSEA) with proven dependability by ESTIMATE analysis. The filtered key genes were analyzed using oncomine, GEPIA, HPA, qRT-PCR, and functional analysis. Patients in a group with a higher stemness and a lower immune infiltration showed a worse overall survival probability, stemness and immune infiltration characteristics of breast cancer progressed in a non-linear fashion. Thirteen key genes related to stemness and immunity were identified and the functional analysis indicated their crucial roles in cell proliferation and immune escape strategies. The qRT-PCR results showed that the expression of *PIMREG* and *MTFR2* differed in different stages of patients. Our study revealed a promising potential for CSC-target immunotherapy in the early stage of cancer and a probable value for *PIMREG* and *MTFR2* as biomarkers and targets for immunotherapy.

## Introduction

Breast cancer (BC) has the highest incidence rate and mortality rate among female malignant tumors, which impacts women’s health significantly (1). It is considered to be heterogeneous depending on molecular subtype and on different stages of cancer progression (2). This heterogeneity poses are changed during treatment, even through various treatment strategies have been developed based on different pathological types (3), especially for the triple-negative BC (4). Thus, further explorations are needed to identify new markers for guiding individualized treatment.

Growing evidence has established the presence of a subpopulation of cancer cells with stem-like properties in most human malignancies, frequently referred to as ‘cancer stem cells’ (CSCs), which possess the long-term ability to initiate and repopulate tumors (5, 6). Diverse mechanisms by which CSCs manage to survive through various strategies including tumor initiation, metastatic reactivation, oncogene- and immune-targeted therapy resistance have been unvealed (4, 7).

Immuno-resistance is one of the main features of tumors that helps them escape immunosurveillance and evade eradication by resisting immunosuppression (8, 9). A myriad of strategies have been discovered in the tumor cells that allow them to circumvent the immune attack, including genetic and epigenetic alterations in the genome of tumor cells that reduce immune recognition and promote protective microenvironment (5, 10).

Evidence has emerged that CSCs have a potential role in regulating their immune characteristics (9, 10), while the molecular mechanism is unclear. In this study, we focused on CSCs in BC. To this end, differentially expressed genes (DEGs) were screened using The Cancer Genome Atlas (TCGA) and Genotype-Tissue Expression (GTEx) databases, mRNAsi index and WGCNA analysis were used in turns to profile the association between stemness of tumor and clinical characters and to identify genes that are related to stemness. Next, we applied immune infiltration analysis to the filtered immune-related genes from stemness and survival-related genes. Finally, the qRT-PCR analysis was used to verify that the expression of *PIMREG* and *MTFR2* was aberrant and diverse in different clinical stages.

## Materials and Methods

### Design and Data Processing of This Study

**Figure 1:**
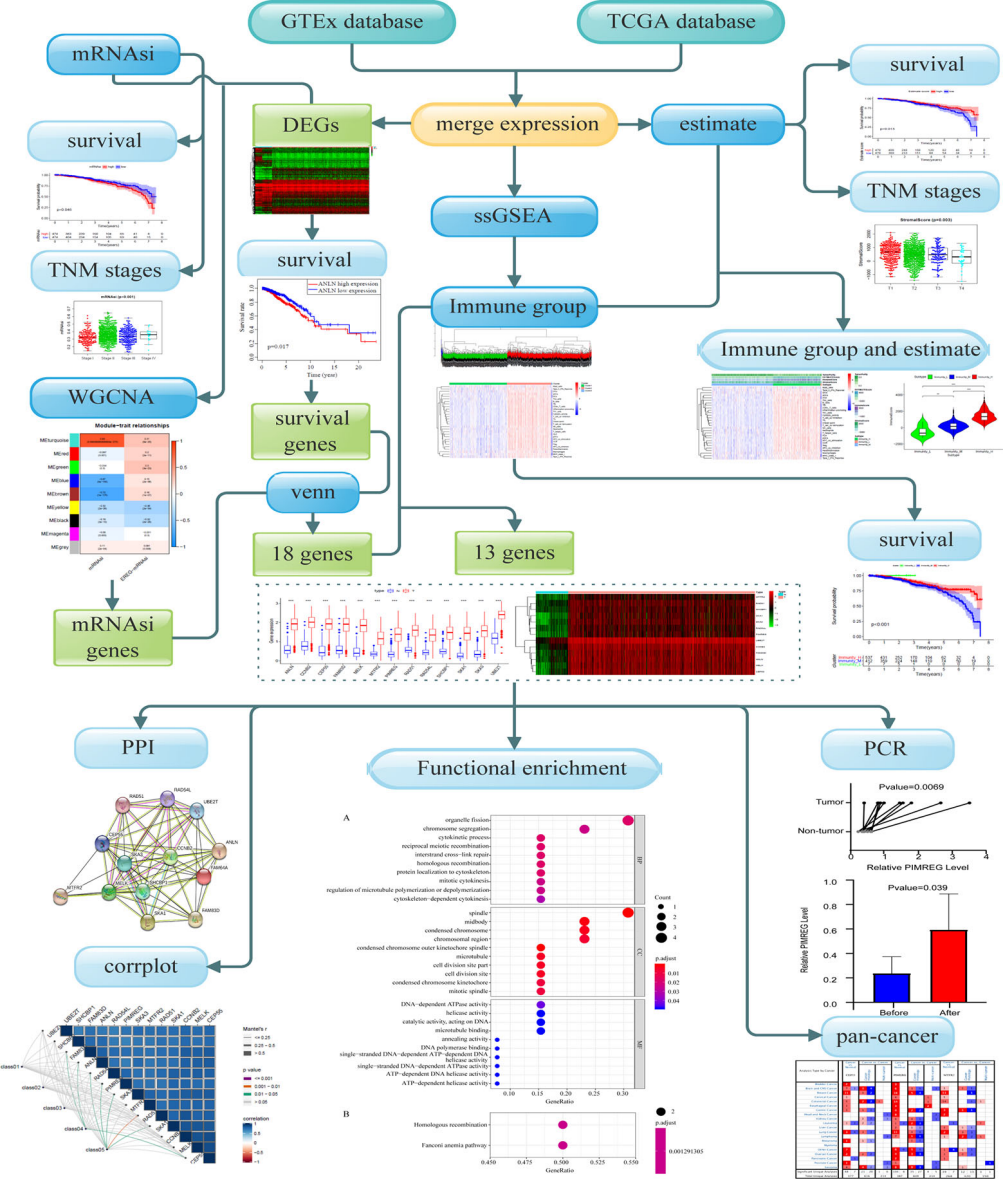
Overall flowchart of key gene identification.

The schematic flowchart of the present study is shown in **Figure 1**. Gene expression and clinical information of patients and healthy individuals was collected from the common database by applying strict filters. Then the computational biology tools were applied, to evaluate stemness and immune characteristics, and biomarkers for patients with BC were revealed. The expression of key genes was evaluated by qRT-PCR.

### Data Acquisition

The comprehensive data of BC including 1,104 tissues of patients and 113 non-tumor tissues was downloaded from the TCGA database (https://portal.gdc.cancer.gov September 26, 2019). Due to the lack of corresponding samples from non-tumor tissues, we utilized GTEx project from where 80 samples of healthy individuals were obtained. RNA-seq results of healthy tissue samples and cancer samples were combined into a matrix file using a merge script in the Perl language (http://www.perl.org/). The mRNAsi were acquired from Tathiane M. Malta et al. (11). The 29 marker gene sets for immune cell types and signaling pathway activation were obtained from Bao X et al. and Liu Z et al. (12, 13).

### Differentially Expressed Genes

The “edgeR” R package was used to screen DEGs of normal breast and cancer samples with the following selection criteria: FDR < 0.05, and |log2 FC| > 1. The values of genes calculated by limma R package, and genes with expression of < 0.5 were deleted. The heatmap and Kaplan-Meier (K-M) curves were drawn.

### WGCNA and Module Merge

Co-expression network was established using the WGCNA R package based on the DEGs. First, RNA-seq data were matched with corresponding mRNAsi and filtered by hierarchical cluster analysis to detect outliers, the Pearson correlation matrix was constructed by correlating coefficient of genes and then transforming them into weighted adjacency matrix using the power function: apq=|cpq|β (cpq=Pearson’s correlation between gene p and gene q, apq=adjacency between gene p and gene q, and β = soft threshold). The best soft threshold (soft threshold = 4) which was selected according to scale independence and mean connectivity for achieving a scale-free co-expression network. The weighted adjacency matrix was then transformed into the topological overlap matrix, DEGs were allocated into different modules for average linkage hierarchical clustering and similar genes were grouped into one module, module dendrogram was drawn with the minimum size (genome), modules with similar heights (cutoff < 0.3) were merged. As a correlation value between genes and sample traits, gene significance (GS) was calculated based on statistical significance, which was determined using the relevant p-values in the linear regression between gene expression and clinical phenotypes (mRNAsi and EREG-mRNAsi). The significant modules related to mRNAsi were selected according to Module significance which was defined as the average GS within the module and revealed the correlation between the module and sample traits.

### Identification of mRNAsi- and Survival-Associated Genes

Module membership (MM) was defined as the correlation between the module’s own genes and gene expression profiles. The key genes associated with mRNAsi were screened by GS and MM defined as cor. gene MM > 0.8 and cor. gene GS > 0.5. The interactive genes between survival-related DEGs and mRNAsi-related genes were finally identified and depicted using venn charts.

### Immune Infiltration Grouping of BC Patients

BC gene set was prepared as a gmt file for further quantitative measurements of the immune activation status. The RNA-seq data of individual cancer samples was transformed into enrichment scores of each immune-related term by Single-Sample Gene Set Enrichment Analysis (ssGSEA) in the R package gsva. Tumors samples with qualitatively different enrichment scores were divided into low, median, high infiltration clusters by using hierarchical clustering analysis in sparcl R package. Results were presented as a color dendrogram and heatmap.

### Tumor Microenvironment (TME) Analysis of BC

The TME scores were calculated based on BC gene set and tumor purity was predicted by using estimate R package. The assessment of TME were divided into four clusters (stromal score, immune score, estimate score and tumor purity) (14, 15). The heatmap and the violin plot were conducted to further explore the relationship between immune groups and TME.

### Identification of Key Genes

All interactive genes associated with survival time and mRNAsi of Venn chart were enrolled for correlation analysis with three infiltration clusters. Genes with no significant difference between immune clusters and mRNAsi were excluded (*P* ≥ 0.05). Correlation analysis and survival analysis of these genes were conducted. The results were shown in box plots, heatmaps, and K-M curves. Oncomine (http://www.oncomine.org), GEPIA (http://gepia.cancer-pku.cn/), and The Human Protein Atlas database (HPA; https://www.proteinatlas.org/) was used to inspect differential expression of key genes between BC and healthy tissues and between different tumors.

### Functional Analysis of Key Genes

Protein-protein interaction (PPI) network analysis was applied to show the relationship between different proteins of these key genes by using String (https://string-db.org/). The functional enrichment analysis was also conducted using clusterProfiler R package to investigate the biological functions of key genes. Gene ontology (GO) functional annotations and Kyoto Encyclopedia of Genes and Genomes (KEGG) pathway enrichment were used in the study with the threshold values: *P* < 0.05, and FDR < 0.05. The results were shown in PPI network graph and dot plots. The relation of co-expressed key genes and clinical characters are analyzed by houyunhuang/ggcor R package.

### Cell Culture

The human breast cancer cell lines, MCF-7 and MDA-MB-231 were obtained from the Chinese Academy of Sciences (Shanghai, China), cell lines were maintained in DMEM (Invitrogen, Carlsbad, CA, USA) with 10% fetal bovine serum (FBS; Sigma-Aldrich, St. Louis, MO, USA), penicillin (100 U/ml), and streptomycin (100 g/ml; Life Technologies, Grand Island, NY, USA) at 37°C with 5% CO_2_.

### Patients

BC samples and corresponding para-cancerous tissues were collected from patients who underwent modified radical mastectomy from January 2020 to March 2020 from the department of endocrine and breast surgery in the First Affiliated Hospital of Chongqing Medical University. Tissues were excised and immediately transferred into liquid nitrogen. All patients were informed and written informed consent were provided. The study was conducted according to the clinical practice guidelines of the International Conference on Harmonization and the Declaration of Helsinki. All patients were diagnosed with triple-negative BC with no evidence of distant metastasis, only patients with Tumor Node Metastasis (TNM) stages II-III were included in the study, certified by two pathologists. BC was divided into two subtypes based on their TNM stages. Requisite clinical data were acquired from hospital records and pathology reports. This study protocol was approved by the ethical committee of Affiliated Hospital of Chongqing Medical University. This study protocol registered with Chinese Clinical Trial Registry (http://www.chictr.org.cn/showproj.aspx?proj=19710; Date of registration: 25/09/2017; Registration number: ChiCTR-PDN-17012784) and was approved by the ethical committee of Affiliated Hospital of Chongqing Medical University (approval number: 2020-119).

### Total RNA Extraction

Total RNA from BC tissues was extracted using an UNIQ-10 column Total RNA Extractio Kit (Sangon Biotech). The RNA concentration and purity were assessed using a SMA4000 microspectrophotometer (Merinton Instrument, Inc) and by RNA electrophoresis with DYY-6C electrophoresis apparatus (Liuyi. Beijing).

### Reverse Transcription and qRT-PCR Quantification

RNA with concentration ranging from 91.84 ng/µl to 1325.94 ng/µl from human BC tumor and para-carcinoma tissues and were reversed-transcribed using a RR047A cDNA synthesis kit (TaKaRa, China). Quantitative PCR was performed for *CEP55*, *MTFR2*, and *PIMREG* using a 2X SG Fast qPCR Master Mix (High Rox, B639273, BBI) with the Step One Plus fluorescence quantitative PCR instrument (ABI, Foster, CA, USA), GAPDH is used for internal control gene. The primers designed by Primer Premier 5.0 are listed as below: MTFR2-F: CTCCTCCACCACTTCCTCCTCAG; MTFR2-R: CGCTCAATTGCACGAAGCTTAACC; PIMREG-F: GAGTGCTTTGGGTGCCGTGTC; PIMREG-R: CCGCCTTGATCGCCGTAATGG; CEP55-F: GTGGGGATCGAAGCCTAGTA; CEP55-R: TCATACACGAGCCACTGCTG.

### Immunohistochemical

The stained images of IHC and the corresponding information in breast tissues and tumors were downloaded from The Human Protein Atlas (https://www.proteinatlas.org). Samples from two patients were included in each group, the IHC procedure were conducted as descripted in the official IHC protocol by Human protein Atlas (https://www.proteinatlas.org/download/IHC_protocol.pdf), in brief, the 4-μm-thick tissue sections from the tissues were pretreated and then stained with the antibodies (*CEP55*: Sigma-Aldrich, HPA023430, *MTFR2*: Sigma-Aldrich, HPA029792, and *PIMREG*: Sigma-Aldrich, HPA043783). Heat Induced Epitope Retrieval (HIER) was used for antigen retrieval. In the Immunohistochemical staining process, slides were first rinsed in wash buffer. then they were Incubated with Ultra V Block, primary antibody, and labeled HRP polymer in order. Next, they were developed in DAB solution, counterstained in hematoxylin, rinsed, dehydrated, and coversliped. The information of stain, intensity and quantity of the photo description were displayed as the results that are shown in The Human Protein Atlas, eg: https://www.proteinatlas.org/ENSG00000129195-PIMREG/tissue/breast#img, to be noted, the phenomenon of the information that is not fully matched with the photo might be caused by the process of manually adjustment: https://www.proteinatlas.org/about/help#2).

### Statistical Analysis

The K-M analysis was applied to show the survival difference of mRNAsi groups and immune clusters. Wilcox analysis was utilized to analyze the difference of mRNAsi between normal and cancer groups. Krudkal-Wallis H test was conducted to reveal the relationship between mRNAsi, infiltration clusters, TME scores, tumor purity and clinical information like TNM stage (*P*<0.05). The beeswarm plots and violin plots were made to visualize the results.

The differences in gene expression between tumor and normal tissues, and para comparison between subgroups were expressed as mean with standard deviations and compared through Student’s paired *t*-test and Student’s *t*-test accordingly by applying GraphPad Prism 8, *P* < 0.05 indicated statistically significant difference.

## Results

### Screening for Survival-Related DEGs

**Figure 2:**
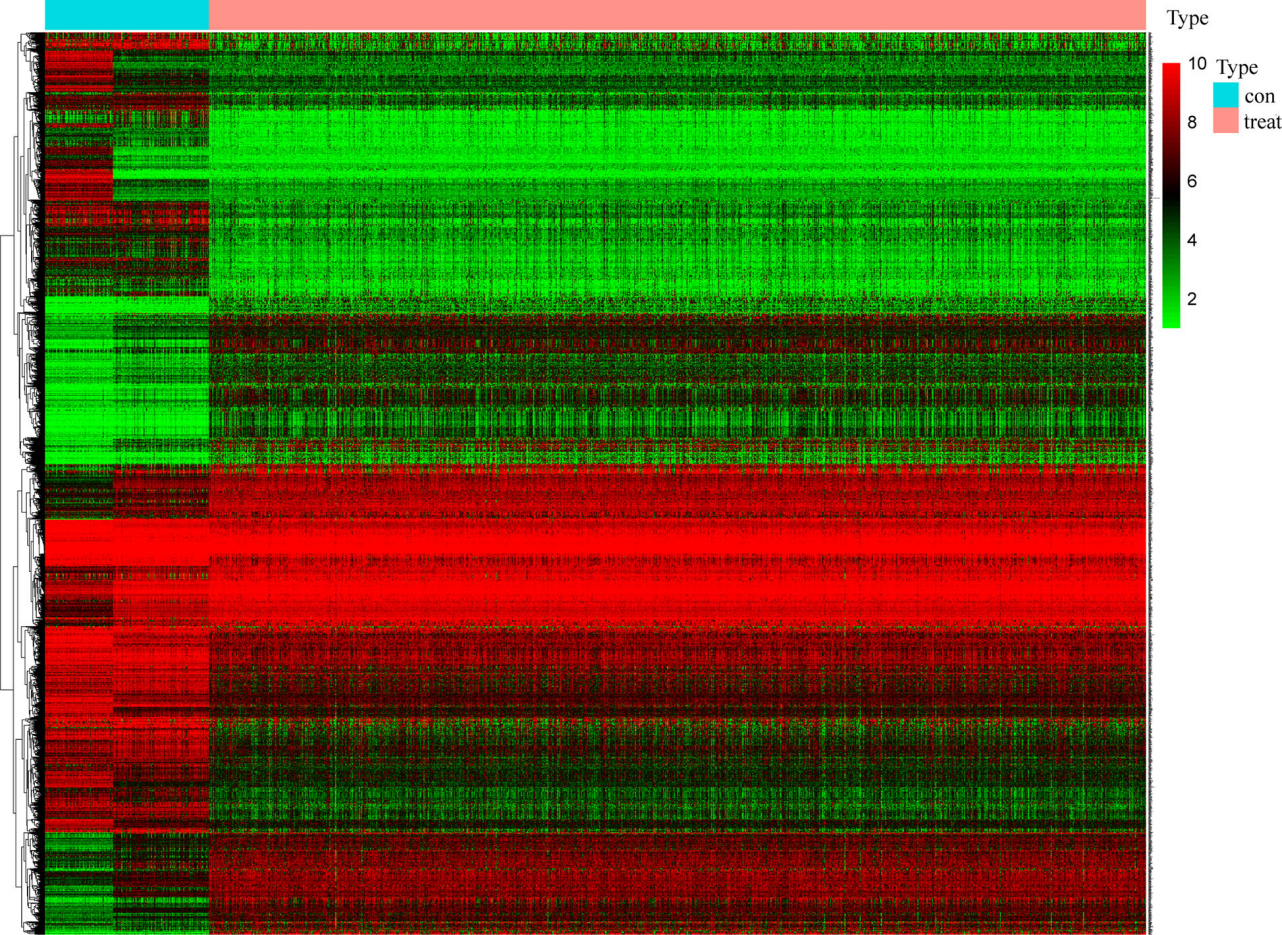
Screening of differentially expressed genes. The heat map of 2261 genes’ expression shows significant difference between normal and tumor samples. Green to red means the gene expression from low to high. The blue band in the top shows normal samples from GTEx and TCGA database and the red band shows cancer samples from TCGA database. (x: samples, y: genes).

Samples for which with RNA-seq and comprehensive information was available were enrolled. A total of 2261 different expression genes were screened with 989 up-regulated genes and 1272 down-regulated genes and compared with normal samples. The results were visualized by heat map (**Figure 2**), in which 212 survival-related DEGs were filtered (including 89 upregulated genes and 123 down-regulated genes).

### Correlation Analysis of mRNAsi and Clinical Characteristics

**Figure 3:**
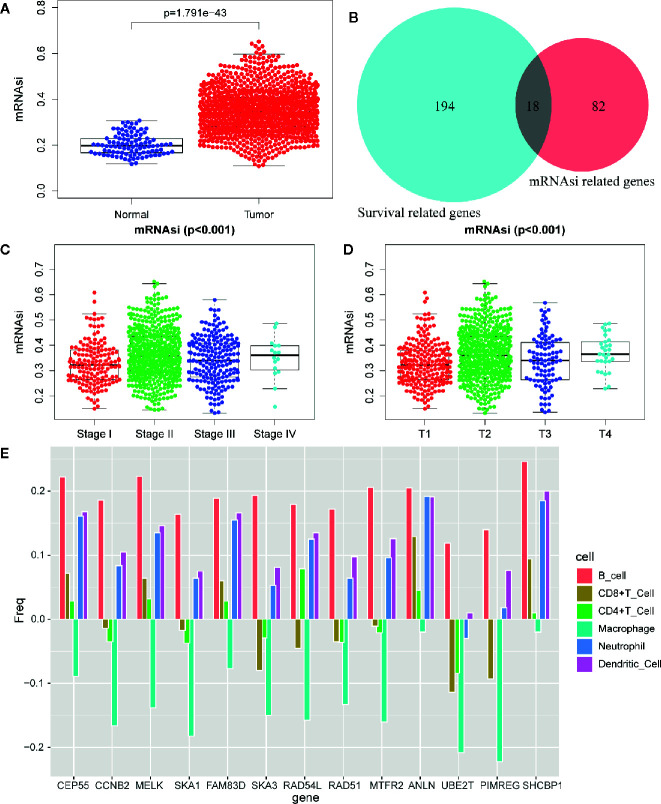
Differentially analysis of mRNAsi in breast cancer. **(A)** The beeswarm plot shows significant difference of mRNAsi between normal (blue dot) and tumor (red dot) samples. The mRNAsi of most tumor samples is higher than normal samples. **(B)** The interactive genes between survival-related DEGs and mRNAsi-related genes. The Venn chat shows 193 survival-related DEGs (blue), 82 mRNAsi-related genes (red) and 18 interactive genes. **(C)** The beeswarm plot shows that TNM stages IV and II have higher mRNAsi. The order of average mRNAsi from high to low is stage IV, stage II, stage III, and stage I. **(D)** The beeswarm plot shows that T4 and T2 have higher mRNAsi. The order of average mRNAsi from high to low is T4, T2, T3, and T1. **(E)** TIMER revealed the correlation of key gene expressions with immune cells (including macrophages, neutrophils, dendritic cells, B cells, CD8+T cells, and CD4+T cells) infiltration level in BC.

**Figure 4:**
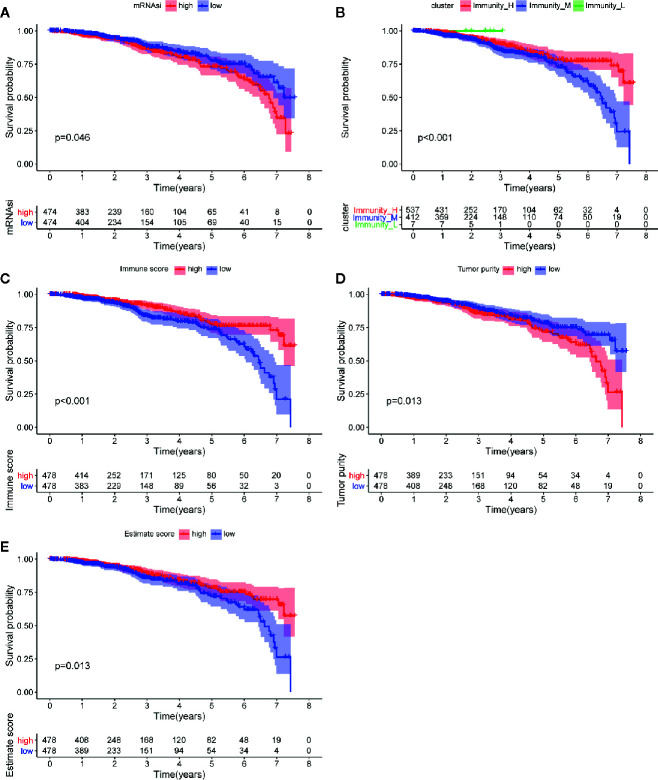
Analysis of prognosis value of mRNAsi and TME. **(A)** The K-M curve shows significant difference between low (blue) and high (red) mRNAsi groups within 5 year-follow-up. **(B)** The K-M curve shows significant differences between low infiltration (green), median infiltration (blue) and high infiltration (red) clusters. **(C)** The K-M curve shows significant difference between low (blue) and high (red) immune score cluster. **(D)** The K-M curve shows significant difference between low (blue) and high (red) tumor purity cluster. **(E)** The K-M curve shows significant difference between low (blue) and high (red) estimate score cluster.

mRNAsi was revealed by Tathiane M. Malta et al. as an index for evaluating the stemness of tumor cells, thus it is also considered as a quantitative description of CSCs. Wilcox analysis showed that the mRNAsi level in cancer group was significantly higher than that of normal group (**Figure 3A**). The K-M curve showed that patients in low mRNAsi group had a longer overall-survival time within 5 year-follow-up (**Figure 4A**). As shown in **Figures 3C, D**, the score for mRNAsi had a rising tendency in a non-linear manner in the progression of BC, to be noted, the dynamic change of stemness of BC tissue from stage II to stage III were inverse comparing to the overall tendency (stage I to stage IV).

### Identification of Significant Modules and mRNAsi-Associated Genes

**Figure 5:**
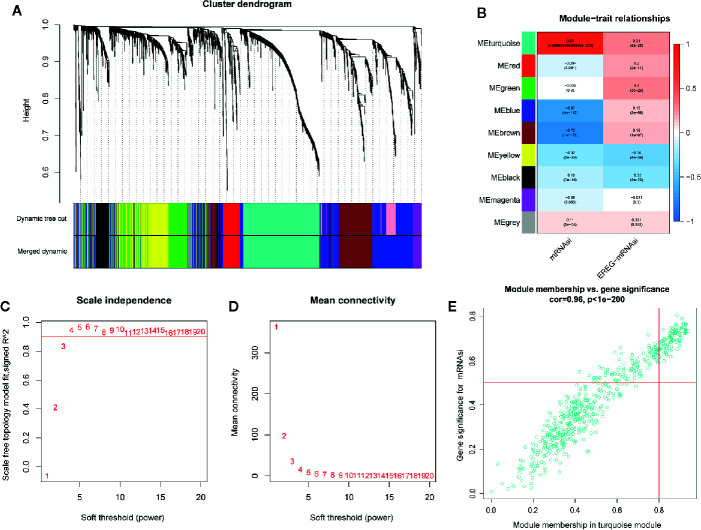
Weighted gene co-expression network of breast cancer. **(A)** Confirmation of hub modules. The branches of the cluster dendrogram represent nine different gene modules with different colors. Every leaf corresponds to a gene. Dynamic Tree Cut corresponds to the original module and Merged Dynamic corresponds to the final module. **(B)** The correlation coefficient shows the relationship between the gene module and the clinical traits (mRNAsi and EREG-mRNAsi). Red corresponds to a positive correlation and blue corresponds to a negative correlation. The corresponding P-value is also annotated. Panels **(C, D)**, respectively, represent for the cluster dendrogram and module-trait relationships of the soft threshold. **(E)** Scatter plot of module eigengenes in turquoise module.

In order to identify biologically significant genes that were associated with BC stemness, a gene co-expression network was conducted by performing WGCNA. DEGs with similar expression patterns were allocated into one module by WGCNA (**Figure 5A**), soft threshold in this study was set at 4 in order to ensure a scale-free network (**Figures 5C, D**), and nine significant gene modules were obtained for subsequent analysis as shown in **Figure 5B**. Next, we explored the correlation between these nine modules and the clinical traits (including mRNAsi and EREG-mRNAsi). The result showed that three gene modules were correlated with mRNAsi (the value of correlation > 0.6), among these, the turquoise module with a positive correlation at 0.83 was most significantly related to mRNAsi, the blue and brown module had significantly negative correlation with mRNAsi (**Figure 5B**). Next, 100 key genes were screened from turquoise module with a threshold defined as cor.MM > 0.8 and cor.GS > 0.5 (**Figure 5E**). Finally, 18 interactive genes were selected from the intersection of 212 survival-related DEGs as well as 100 mRNAsi-related genes.

### Immune Phenotype Landscape in the TME of BC

**Figure 6:**
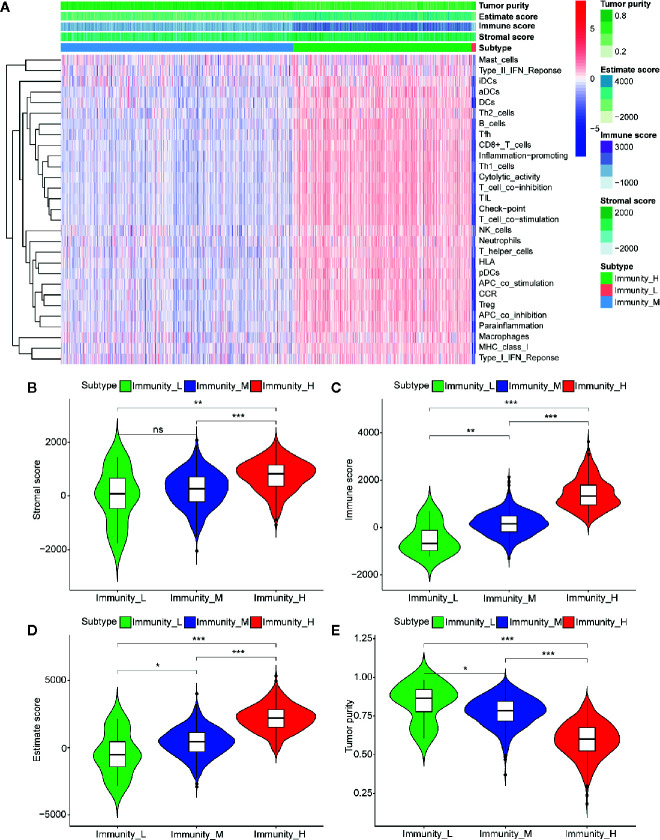
Three infiltration clusters and TME relevance of BC patients after using ssGSEA according to 29 marker gene sets. **(A)** 1069 BC samples are divided into high infiltration (red band), median infiltration (green band), and low infiltration clusters (blue band). The assessment of infiltration clusters conducted by TME are clustered into stromal score **(B)**, immune score **(C)**, and tumor purity **(E)** estimate score **(D)**. (ns, p > 0.05; *p < 0.05; **p < 0.01; ***p < 0.001).

**Figure 7:**
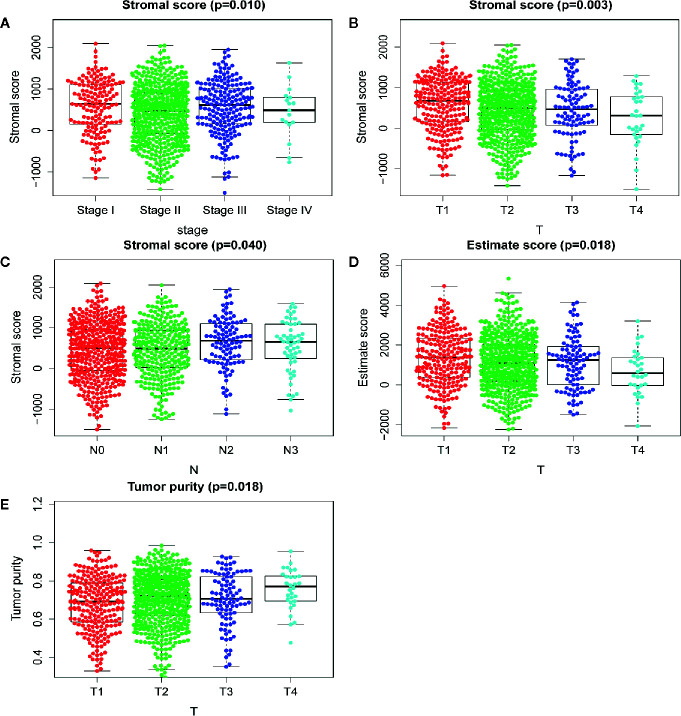
Analysis of clinicopathological characteristics of mRNAsi and TME. **(A)** The beeswarm plot shows TNM stage I and stage III have higher stromal score. The order of average stromal score from high to low is TNM stage I, TNM stage III, TNM stage II, and TNM stage IV. **(B)** The beeswarm plot shows that T1 has a higher stromal score. The order of average stromal score from high to low is T1, T2, T3, and T4. **(C)** The beeswarm plot shows that N2 and N3 have higher stromal score. The order of average stromal score from low to high is N1, N0, N3, and N2. **(D)** The beeswarm plot shows that T1 and T3 have higher Estimate score. The order of average Estimate score from high to low is T1, T3, T2, and T4. **(E)** The beeswarm plot shows that T1 and T3 have lower tumor purity. The order of average tumor purity from low to high is T1, T3, T2, and T4.

The immune cell infiltration status of each sample was assessed by ssGSEA and transformed into enrichment scores based on its transcriptomes according to 29 marker gene sets. All 1104 BC samples were allocated into three hierarchical clusters (low infiltration: 11 patients; intermediate infiltration: 618 patients; and high infiltration: 475 patients) (**Figure 6A**). ESTIMATE method was then applied to testify the reliability of this model, which evaluated the conditions of cellular composition in each sample by multiple indicators, including stromal score, immune score, tumor purity, and an ESTIMATE score that is regarded as sum of the first three scores. The results showed that patients in high infiltration cluster had higher stromal score, higher immune score, lower tumor purity, and higher overall ESTIMATE score (**Figures 6B–E**). Survival analysis was also applied to assess the of prognosis value of ESTIMATE scores. As shown in **Figures 4B–E**, high immune score, low tumor purity, and high ESTAMATE score had a longer overall-survival time. As shown in **Figure 7**, stromal score and estimate score showed a downward tendency in a non-linear manner in the tumor progression, while estimate score showed a rising tendency in a non-linear manner in the progression of Lymph Node. To be noted, the change of stomal score from stage II to stage III and ESTAMATE score from T2 to T3 showed inverse correlation compared with the global trend. The Stromal score of N2 reaches the top.

### Key Genes Identification and Correspondence Functional Analysis

**Figure 8:**
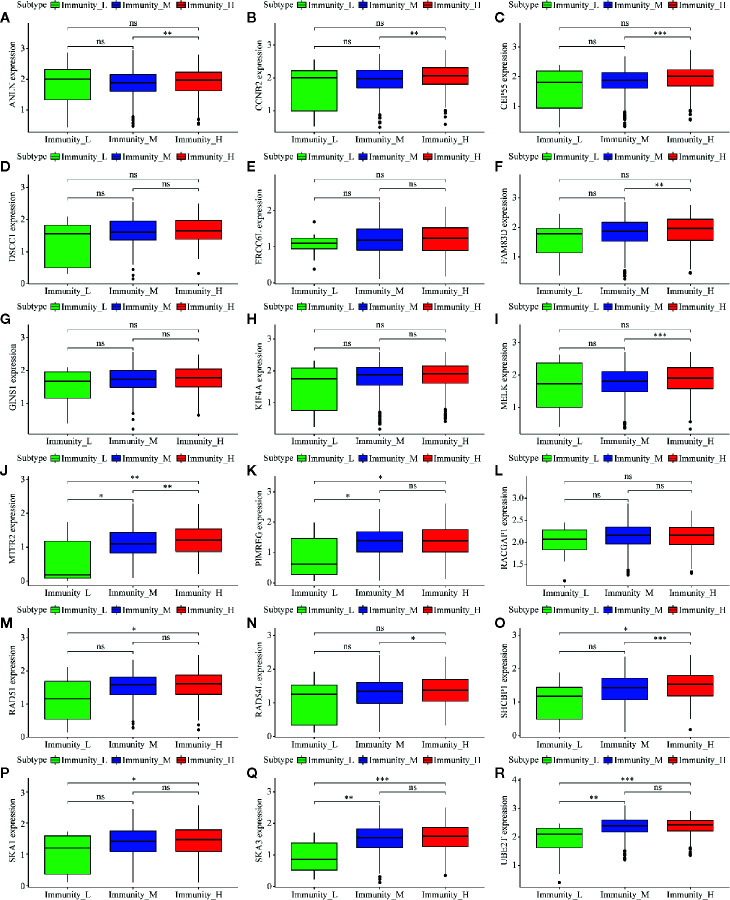
Correlation analysis between 18 mRNAsi-related genes and infiltration clusters. **(A)** to **(R)** shows the expression levels of the mRNAsi-related genes in different clusters.The boxplots show there is no statistical difference on the expression of DSCC1 **(D)**, ERCC6L **(E)**, GINS1 **(G)**, KIF4A **(H)** and RACGAP1 **(L)** between 3 infiltration clusters. While the left genes (ANLN, CCNB2, CEP55, FAM83D, MELK, MTFR2, PIMREG, RAD54L, RAD51, SHCBP1, SKA1, UBE2T, and SKA3) are analyzed with a difference between infiltration clusters. (ns, p > 0.05; *p < 0.05; **p < 0.01; ***p < 0.001).

**Figure 9:**
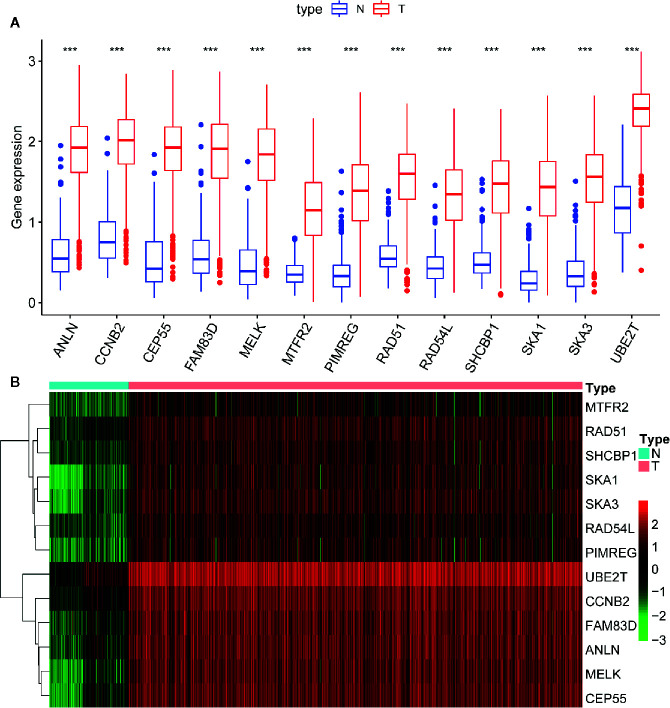
Difference analysis of the gene expression between normal and tumor samples. **(A)** The boxplot shows significant difference of gene expression between normal and tumor sample. The expression of these 13 genes in tumor sample are higher than in normal samples. **(B)** The heatmap shows the expression change of genes from normal (blue band) to tumor (red band) samples. Green to red means gene expression from low to high. ***p < 0.001.

**Figure 10:**
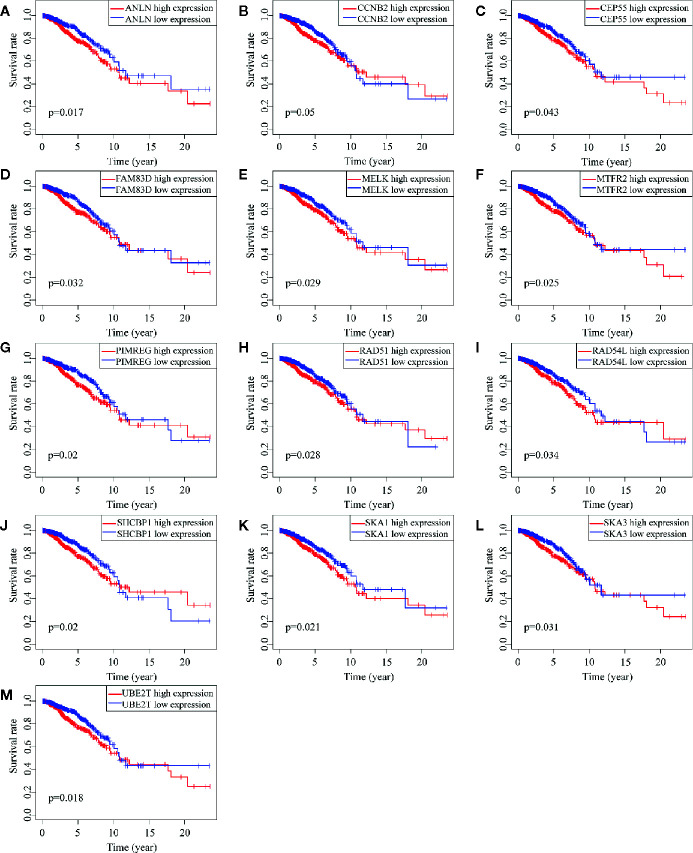
Survival analysis of 13 key genes. **(A–M)** shows the different survival time of patients between low and high expression of 13 genes. All the K-M curve shows significant difference that low expression of these genes has a longer OS.

Of 18 mRNAsi-related genes that were analyzed for correlation with three immune cell infiltration clusters, 13 key genes were finally enrolled (**Figure 8**). There was statistical significance of the expression of *ANLN*, *CCNB2*, *CEP55*, *FAM83D*, *MELK*, *MTFR2*, *PIMREG*, *RAD54L*, *RAD51*, *SHCBP1*, *SKA1*, *UBE2T*, and *SKA3* within three immune cell infiltration clusters. As shown in **Figure 9**, the expression levels for all 13 key genes were higher in tumor samples than in the normal samples. The K-M curves showed that patients with higher level of expression of these genes had a poor overall survival time (**Figure 10**). The results indicated that these 13 key genes were associated with mRNAsi and immunization as good prognostic factors.

**Figure 11:**
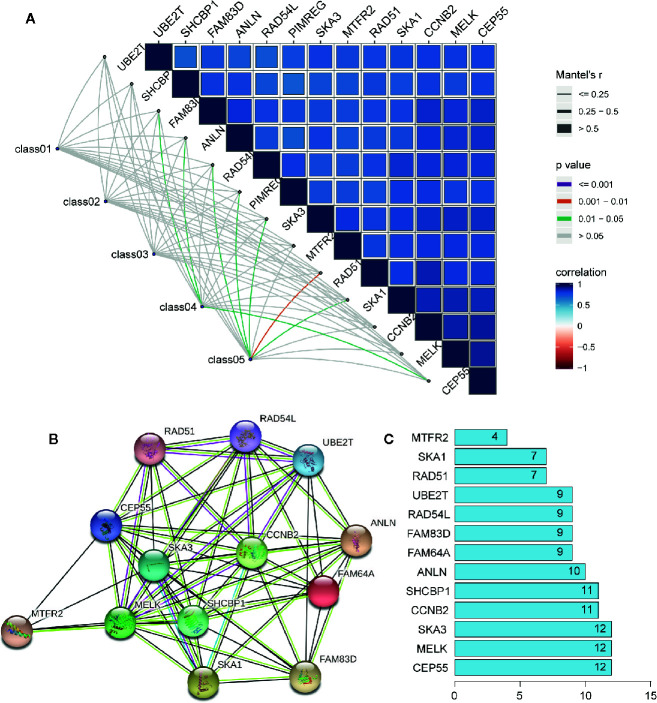
The interaction between key genes. **(A)** Mantel analysis of co-expression of 13 key genes and clinical information, Class01 to Class05, respectively, represent the survival times and status, TNM stage, tumor microenvironment, mRNAsi and EREG-mRNAsi, and immune group. **(B)** PPI network analysis of 13 key genes. Thickness of the solid line represents the strength of the relationship. **(C)** Histogram shows the number of nodes of 13 key genes in PPI network.

**Figure 12:**
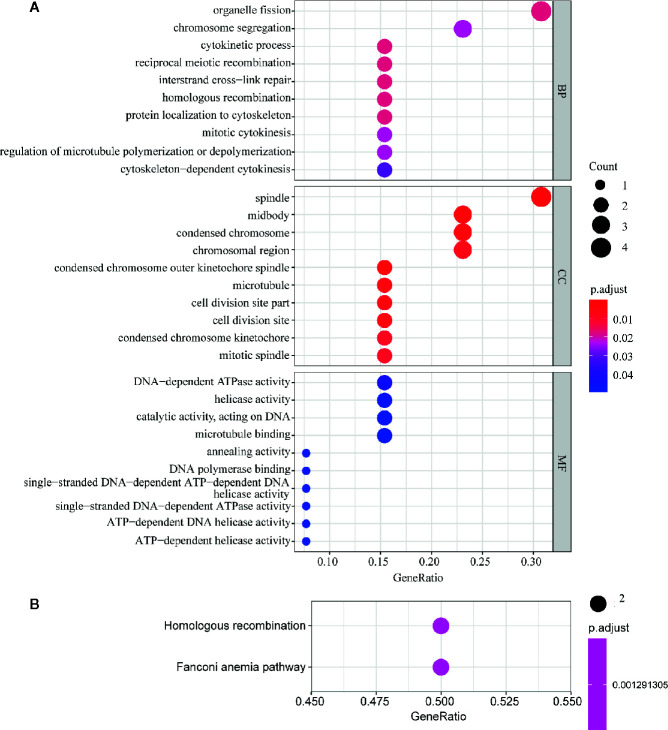
GO and KEGG pathway enrichment analysis of key genes. The size of the circle represents the number of genes and the y-axis shows the GO and KEGG pathway terms. The redder the color the higher the value of p. Molecular Function (MF); Biological Process (BP) and Cellular Component (CC). **(A)** Enrichment of Gene Ontology (GO) analysis. **(B)** Enrichment of Kyoto Encyclopedia of Genes and Genomes (KEGG) pathway analysis.

**Figure 13:**
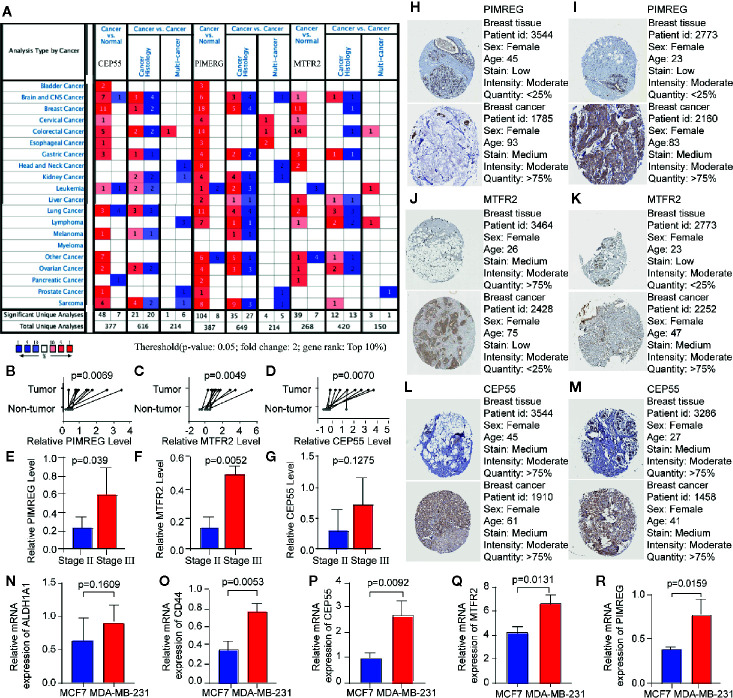
The mRNA expression of key genes in multiple cancers and in BC patients. **(A)** The expression of key genes in pan-cancer scale is evaluated by Oncomine, the number in the table cell is determined by the number of data that meet thresholds. The color depth represents the gene rank. The red cells suggest that the expression levels of key genes are relatively higher in tumor tissues, while blue cells imply the opposite. **(B**–**G)** The result of qRT-PCR compared the differential expression of key gene in tumor and para-carcinoma tissue of patients, the expression between patients in stage II and stage III group is also evaluated. **(H**, **J**, **L)** The immunohistochemical staining revealed the staining patterns of *PIMREG*, *MTFR2* and *CEP55* between breast cancer samples and normal mammary samples. **(I**, **K**, **M)** *PIMREG*, *MTFR2* and *CEP55* expression profile of breast cancer patients from GEPIA database. **(N–R)** relative expression level of ALDH1A1, CD44, CEP55, MTFR2, and PIMREG in MCF7 and MDA-MB-231 cell lines.

In order to further explore the molecular function of the 13 key genes identified, PPI and enrichment analysis were conducted. As shown in **Figures 11B, C**, *CEP55* proteins had one of the highest node number (node number is 12). The clinical significance of these co-expressed key genes is shown in **Figure 11**. GO functional enrichment analysis indicated these genes were related to cell proliferation, such as organelle fission, chromosome segregation, spindle, midbody, and chromosomal region (**Figure 12A**). The results of KEGG showed the top enriched terms were homologous recombination and Fanconi anemia pathway and indicated a close correlation with DNA damage and repair (**Figure 12B**). The result of TIMER showed that most of the key genes had strong correlation with B lymphocyte and dendritic cells’ function (**Figure 3E**). Through differentially expression analysis of pan-cancer and normal samples by Oncomine and protein expression scan by HPA, it is noted that the evaluation of protein expression score was based on immunohistochemical, which were manually scored with regard to staining intensity including negative, weak, moderate or strong, and fraction of stained cells including <25%, 25–75%, and >75%, next the combination of intensity and fractions was converted into an protein expression level score and listed as follows: weak combined with either 25 - 75% or 75% and moderate <25% were shown as low, which included PIMREG and MTFR2 in breast tissue; moderate combined with either 25 - 75% or 75% and strong <25% were shown as medium, which contained the rest of the images. Protein expression values were also manually adjusted by expert annotators when necessary. It was found that *PIMREG*, *MTFR2*, and *CEP55* were overexpressed in BC and also in many other cancers (**Figures 13A, H–M**).

### Validation of Key Genes Expression Using qRT-PCR

The expression levels of *PIMREG*, *MTFR2*, and *CEP55* were assessed by qRT-PCR, which showed a significantly higher level of expression for these genes in BC samples compared with that in corresponding adjacent breast samples. We also investigated the relationship between gene expressions and TNM stage. Significantly different expression levels of *PIMREG* and *MTFR2* were identified between TNM stage II group and stage III group as shown in **Figures 13B–G**. Next, we investigated whether expression levels of PIMREG, CEP55, and MTFR2 is related to the expressions of BCSC markers in breast cancer cell lines. Two breast-cancer cell lines were compared, including MCF7 and MDA-MB-231, which are respectively known as negative and positive cell lines for BCSC markers [47]. Two well-known breast cancer stem-cell markers, CD44 and ALDH1 family member A1, were applied to verify this result. RT-PCR indicated that the expression level of PIMREG, CEP55, and MTFR2 are relatively high in BCSC-marker-positive cells (MDA-MB-231) in comparing with BCSC-negative cells (MCF-7).

## Discussion

Typical treatments for BC besides surgery include endocrine therapy, chemical therapy, and radiotherapy; these have led to increased survival rates in majority of patients since most of the tumor cell express and respond to receptors for estrogen and progesterone receptor. However, as the most aggressive subtype, triple-negative BC have limited treatment options and worse prognosis. The presence of CSCs was discovered as one of the main causes for treatment resistance and there is lack of targets for CSCs in advanced cancers. In fact, one of the main manifestations in the cancer progression is the gradual loss of the differentiated phenotype and gaining stem cell-like characteristics (5). Evidence showed that the frequency of CSC is increasing with tumor progression in multiple solid cancer types (16); however, this process is non-linear in BC due to the dynamic negative feedback control of tumor cell populations and high heterogeneity within cells in BC tissue (17). Therefore, it is crucial to observe the dynamic change of the stemness characteristics in the progression of BC. In this study, we discovered that the mRNAsi score was the lowest in stage I and reaches the highest in stage IV, which matched with the data published by earlier researchers. To be noted, tumor reached the first peak in stage II (and T2) and decreased in stage III (and T3) of the stem cell characteristics, this provided an insight into a specific timeline for detecting the vitality of breast CSC (17).

Diverse mechanisms have been unraveled that could help CSCs to survive under hostile environment, including enhancing DNA-repair capacity (18), among which are overexpression of multifunctional efflux transporters and aberrant activation of developmental pathways (19, 20). We applied mRNAsi-based WGCNA and identified the module that was associated with BCSC characteristics. Functional annotations of the module were primarily associated with cell cycle control, cell proliferation characteristics, and DNA-damage repair.

Immunotherapy has been developed rapidly in recent years due to the deepened acknowledgement of the immune system evasion by tumors. Developments, such as immune checkpoint inhibitors and receptors, have been made for the treatment of aggressive cancer. In order to observe the potential immune target in the CSC, we depicted the immune landscape of BC by clustering all patients into three clusters of different immune infiltration degrees. Our immune landscape revealed that T cell mediated immune response and innate immune response may lead the anti-tumor effect, further results from TIMER have confirmed these findings and provided a more specific profile of immune include dendritic cell and B lymphocyte. This provided a prospect and reminder in the direction of CSC-target immunotherapy since there have been successful attempts of generating CSC-primed T cells *in vitro* that showed targeting of CSCs after adoptive transfer *in vivo* (21). It has also been demonstrated that significant anti-CSC immunity was induced by dendritic cell vaccine basing on CSC both *in vitro* and in immune-competent hosts (8, 22). B lymphocytes were recently found to play a pivotal role in the cancer immune by being an assistant for T lymphocyte (23).

We also testified the reliability of the immune landscape by applying ESTIMATE analysis, as shown by someone, the relevance of breast CSC markers may vary according to the heterogeneity of the TME (24), where contains multiple types of cells, such as fibroblasts, endothelial cells, and mesenchymal stem cells, they are thought to have an affection to breast CSCs survival by secreting signaling molecules. Our results showed that patients in the high-infiltration cluster had the highest immune cell, highest stromal cell proportion, and lowest tumor purity compared with those in patients in the low- and intermediate-infiltration clusters, which is in agreement with previous studies. The change the stromal score from stage II to stage III and the estimate score from T2 to T3 also showed inversion, which synchronized with the stemness character. We found samples in stage IIIA tend to have highest stromal scores (N2), higher estimate scores (T3) and lower mRNAsi while samples in stage II tended to have lower stromal scores (N0-1), lower estimate scores (T2) and higher mRNAsi. These results indicated that the immune-like activity enhanced and likely validly regulated the stemness of BC cells in stage IIIA compared to stage II. The relationship between immune characteristics and stemness needs to be explored and the genes related to both stemness and immune properties are promising research targets.

As Pan Q et al. stated, identification of specific antigens or genetic alterations in CSCs may provide more specific targets for immunotherapy (25). In order to uncover the epigenetic regulations of BCSC have utilized to survive under immune surveillance, the stemness-related genes were assessed under different immune infiltration clusters, 13 stemness-related genes were found to expressed differently, expression level of these genes were significantly upregulated in tumor samples and associated with poorer overall survival and progression in BC. Among these, RAD51 is a protein encoding gene that is recruited to the perturbed replication DSBs and forks sites and respectively blocks the exonuclease activity of MRE11 on DSB repair and on the replicated genome and eventually limit self-DNA accumulation in the cytosol, this process also prevents the initiation of innate immune signaling mediated by STING (26), which brought an insight to the potential relationship between stemness and immune escape mechanisms in the tumor cell.

**Figure 14:**
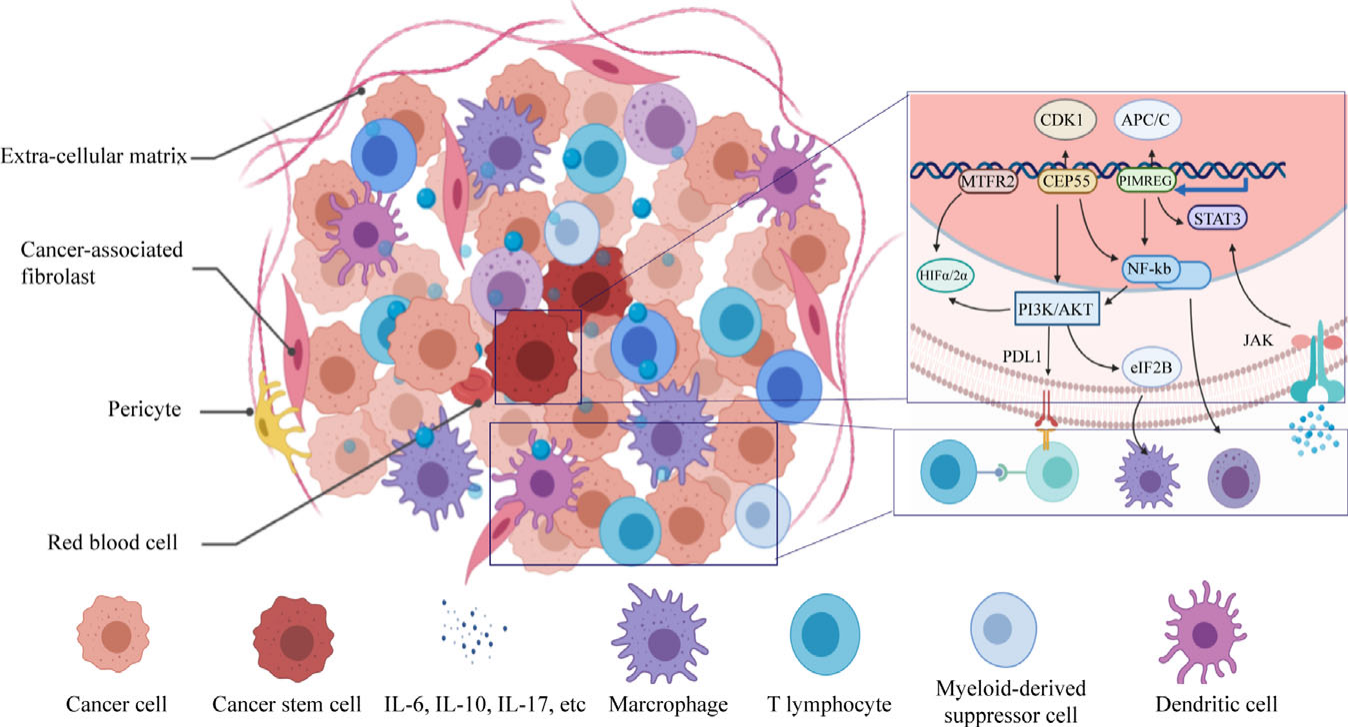
Schematic diagram of immuno-resistance profile of the CSCs.

In order to observe the relationship between the expression of key genes and clinical characteristics of patients, we applied qRT-PCR to evaluate the expression of *PIMREG*, *MTFR2*, and *CEP55* (**Figure 14**). Results have shown that these genes were highly expressed in BC tissue, these results indicated that the selected key genes may become therapeutic targets of BC, to be noted, *PIMREG*, *MTFR2* were shown to have higher expression in TNM stage II compared with stage III, this indicate that the selection of preferable timing is rather crucial, for both the stemness of the tumor cells and immune infiltration characters of tumor tissue are in the phases of competition. *PIMREG* is commonly known as a cell cycle promoter of hypoxic fetal cardiomyocytes (27). It is known as a key gene involved in exogenous infections include suppurative periapical periodontitis (28) and paragonimiasis (29). It has been discovered that PIMREG promoted the aggressiveness of BC by disrupting the NF-κB/IκBα feedback loop (30). To be noted, NF-κB activation was proven to be involved in the tumor associated macrophages-mediated tumor growth in human pancreatic ductal adenocarcinoma (31). *PIMREG* is known to positively regulate STAT3 activity to promote cell differentiation and shown to be associated with poor survival in the BC and pancreatic cancer (31–34). It has been proven that both anti- and proinflammatory cytokines signal through associated receptor/JAK complexes and result in the phosphorylation of STAT3 (35). *CEP55* protein is required for membrane fusion of cytokinesis through the inhibitory of cyclin-dependent kinase 1 phosphorylation (36, 37), *CEP55* was found to inhibit apoptosis of human glioma cells *via* the PI3K/Akt signaling pathway, the PI3K/Akt pathway is a crucial pathway in the immune escape, it promotes the progression of BC growth by suppressing NK cell cytotoxicity through eIF2b (38), also, the inhibition of PI3K in lung cancer downregulated PD-L1 expression (39). *MTFR2* is known as a protein that belongs to the MTFR family, it has been found that the *MTFR2* was upregulated in BC and associated with poor survival of BC patients (40). MTFR2 alters glucose metabolism through activating HIF1α and HIF2α (EPAS1) in the BC cell lines (40, 41), the stability of which is also controlled by PI3K/AKT signaling axis (42). *MTFR2* was also identified as an activator of TTK promoter in glioma stem-like cells (43). It is also reported to play a role in mitochondrial aerobic respiration essentially (43) and the promotion of mitochondrial fission (41). One of the strengths of the immune-therapies is the ability to target multiple antigens, which makes these approaches perfectly suited for targeting heterogenous CSC populations (44). *PIMREG* and *MTFR2* are considered as potential target genes in CSC population that are required for effective immune targeting, the pan-cancer analysis implies that these genes may also play important roles in other tumors.

## Conclusion

We identified 13 key genes related to stemness and immune escape and the stemness and immune escape degree of BC were increased non-linearly in the early stage of BC progression. *PIMREG* and *MTFR2* are considered as effective diagnostic markers and potential targets for therapy. The development of stemness is involved in the regulation of both innate- and acquired-immune microenvironment.

## Data Availability Statement

The raw data supporting the conclusions of this article will be made available by the authors, without undue reservation.

## Ethics Statement

The studies involving human participants were reviewed and approved by The First Affiliated Hospital of Chongqing Medical University. The patients/participants provided their written informed consent to participate in this study. Written informed consent was obtained from the individual(s) for the publication of any potentially identifiable images or data included in this article.

## Author Contributions

Conception and design: JXT and LW. Acquisition of data: JXT and YW. Analysis and interpretation of: LW. Writing, review and/or revision of manuscript: LW, JL, and JJT. Administrative, technical or material support: XY. Study supervision: LW and JL. All authors contributed to the article and approved the submitted version.

## Conflict of Interest

The authors declare that the research was conducted in the absence of any commercial or financial relationships that could be construed as a potential conflict of interest.

## Abbreviations

BC, Breast cancer; CSCs, Cancer stem cells; DEGs, Differentially expressed genes; TCGA, The Cancer Genome Atlas; GTEx, Genotype-Tissue Expression; K-M, Kaplan-Meier; GS, Gene significance; MM, Module membership; ssGSEA, Single-Sample Gene Set Enrichment Analysis; TME, Tumor microenvironment; PPI, Protein-protein interaction; GO, Gene ontology; KEGG, Kyoto Encyclopedia of Genes and Genomes; TNM, Tumor Node Metastasis.
